# Nurses’ and Physicians’ Responses to a New Active Antimicrobial Stewardship Program: A Two-Phase Study of Responses and Their Underlying Perceptions and Values

**DOI:** 10.34172/ijhpm.2022.6557

**Published:** 2022-05-18

**Authors:** Jacob Strahilevitz, Shaul Oreg, Ran Nir Paz, Lilach Sagiv

**Affiliations:** ^1^Department of Clinical Microbiology and Infectious Diseases, Hadassah-Hebrew University Medical Center, Jerusalem, Israel.; ^2^Faculty of Medicine, The Hebrew University, Jerusalem, Israel.; ^3^School of Business Administration, The Hebrew University, Jerusalem, Israel.

**Keywords:** Antimicrobial Stewardship, Personal Values, Change Attitudes, Psychological Situations, Handshake Stewardship

## Abstract

**Background: ** Successful implementation of an antimicrobial stewardship program (ASP) depends on staff members’ response to it. We introduced at the Hadassah Medical Center in Israel a significant change to our long-standing handshake ASP. As before, the new ASP involved a dialogue between the treating physician and the infectious disease physician over the appropriate antibiotic therapy. The main change was that the infectious disease physician’s decision was now integrated into the patient’s electronic medical record (EMR). Our purpose in this study was to uncover the concerns and expectations of physicians and nurses towards the new ASP, before and after its implementation, and link these with their basic perceptions of the ASP and their personal values.

**Methods: ** We used open-ended questions and Likert-type scales to study staff members’ personal values, basic perceptions of the new system, and attitudes towards it, both before (N = 143), and one year after (N = 103) the system’s implementation. Relationships of the system’s perceptions and personal values with attitudes toward the system were tested using correlations and multiple regression analyses.

**Results: ** Prior to its implementation, physicians and nurses had multiple concerns about the new ASP’s demandingness and inefficiency and its threat to physicians’ autonomy and expertise. They also had positive expectations for benefits to the hospital, the patients and society. A year later, following the system’s implementation, concerns dissipated, whereas the perceived benefits remained. Moreover, staff members’ attitudes tended to be more positive among those who value conformity.

**Conclusion: ** Introducing new ASPs is a challenging process. Our findings suggest that hospital staff’s initial concerns about the new ASP were primarily about its ease of use and demandingness. These concerns, which diminished over time, were linked with perceived satisfaction with the system. Conformity values had an indirect effect in predicting satisfaction with the system, mediated by perceptions of the system as straightforward.

## Background

**Key Messages**

** Implications for policy makers**

- Antimicrobial stewardship programs (ASPs) are a key strategy to improve prescription of antimicrobial agents and to reduce the development and selection of antibiotic- resistant bacteria. It requires healthcare professionals from multiple disciplines to change behavior, and may be perceived as loss of autonomy. Our findings highlight the importance of clearly articulating ASPs’ benefits, and alleviating concerns about their demandingness as a means of improving these programs’ implementation.
- Highlighting the institution’s involvement in introducing ASPs could strengthen staff members’ buy in to the new system.
- The design of ASPs and the plan for their implementation process should account for differences recipients’ occupation and personal values.

** Implications for the public**

Antimicrobial stewardship is at the core of the infectious diseases (ID) practice globally. The collaboration of physicians and nurses, who directly responsible for the patients, is a prerequisite for its successful implementation. It is therefore essential to understand physicians’ and nurses’ attitudes towards the new program, as well as their underlying perceptions and motivations. Recently, we introduced a major change the handshake stewardship processing; it involved greater shared patient responsibility, but ID physicians’ decisions became final, thus preventing the dispensing of non-authorized antibiotics. We uncovered a variety of concerns and expectations about the new stewardship program, and linked these with staff members’ basic perceptions of the program and their personal values. Our findings highlight the role of clear and early communication of the new program’s advantages for multiple stakeholders, including both patients and the hospital, as a means of facilitating a smooth program implementation and diffusion.

Antimicrobials have a unique place in patients’ therapy because alongside their benefits, their use also has the potential for collateral damage in the form of the selection of drug-resistance and their unwanted spread to others and to the environment. The rise of antimicrobial resistance has driven the initiation and integration of antimicrobial stewardship programs (ASPs) in many hospitals worldwide. The Centers for Medicare & Medicaid Services of the Department of Health and Human Services in the United States, as well as other regulatory agencies, require all acute-care hospitals to develop and implement an ASP.^1^ These programs aim to optimize antimicrobial use via a formalized strategy which ensures that antimicrobials are used appropriately.^2^ Prospective audit and feedback improve the judicious use of antibiotics and are recommended as core components of any stewardship program.^3^ For over four decades, we have used at our hospitals the handshake antimicrobial stewardship method, which is distinguished from other methods by (1) lack of prior authorization, (2) prospective audit by the infectious diseases (ID) physicians with (3) in-person feedback to the prescribers. For the latter, the ID physicians who provided consults also authorized pharmacies to dispense specific antibiotics, or stop dispensing altogether (Mervyn Shapiro, personal communication). The process is designed primarily for those cases in which the ID consultation involved the modification or termination of antibiotic treatment, but ID physicians welcomed additional consults for other patients with suspected or confirmed infection.^4^ Physicians were, however, able to adhere to their prior treatment decisions.

 Recently, a major change in the implementation of ASP has been introduced, consisting of the integration of the ASP service into patients’ electronic medical record (EMR). This modification implies stronger shared patient responsibility, whereby the prescriber and ID physician need to reach consensus about the patient’s treatment plan, which is then recorded in patients’ EMR, thus making it final and preventing the dispensing of non-authorized antibiotics. Consequently, dispensing of antimicrobials by the pharmacy to the wards was based on consumption, and nurses were not required to obtain permission to administer treatment without ID approval.

 Although such changes are mandatory, their success nevertheless depends on stakeholders’ reaction to them, specifically the response of the physicians and nurses.^5^ We know that ASPs elicit a myriad of responses, both positive and negative, which are important to understand and predict for more successfully implementing such programs. Previous research identified several factors that affect program success. These include different specialty cultures, such as those of surgeons, who value individualism, versus medical teams who prefer collectivism,^6,7^ the nature of the relationship between ID physicians and prescribers,^8^ the degree to which the ASP is tailored to meet local needs,^9^ and prescribers’ familiarity with ASP practices.^10^ Beyond these insights, it is also important to uncover the psychological factors that underlie stakeholders’ responses. Specifically, our interest was in the perceptions and values that underlie these responses. Given what we know about the relationships between perceptions, values, and people’s attitudes and behaviors in general and in the work context,^11,12^ understanding these factors should be useful for the effective implementation of ASPs.^4,13,14^ To this aim, we conducted a bottom-up analyses of nurses’ and physicians’ responses through a survey, including both open-ended and Likert-type scales. Specifically, we aimed to identify nurses’ and physicians’ main concerns about, and expectations from the new drug administration process and test the relationships of these concerns and expectations with nurses’ and physicians’ personal values and the manner in which they perceive the new system.

## Materials and Methods

###  Setting

 The Hadassah-Hebrew University medical centers, in Jerusalem, Israel, is a 1150-bed, tertiary-care teaching hospital with two campuses. The centers include 1070 physicians and 1800 nurses. There are approximately 450 000 patient days and 100 000 admissions annually, with 20 000 patient-unique orders of antibiotic treatment, generating approximately 7500 yearly ID consultations on top of other ID consultations. Consults are carried out each weekday by 3-4 physicians.

###  Study Procedure

 The integration of the new ASP in the EMR was done in 25 out of the 45 adult inpatient wards, following a presentation of the system to the physicians and nurses in each ward. During this meeting, physicians and nurses were asked to complete questionnaires in two phases, the first before implementation, and the second one year later. In Phase 1 the questionnaires were administered during staff meetings, immediately prior to the implementation of the new antibiotic approval system, after providing an explanation of the changes and their advantages. In Phase 2, the staff were asked to complete a second questionnaire online. Due to the anonymous nature of the questionnaire, an unknown proportion of the respondents in Phase 2 had not participated in Phase 1.

 The Phase 1 questionnaire included: (1) two open-ended questions about the positive and negative aspects that staff members identify in the new system (“Please describe the positive aspects of the new system”; “Please describe the negative aspects of the new system”), (2) an abbreviated measure of basic motivations (personal values, a ten-item scale, see Supplementary file 1), and (3) a situation perception scale (18-item scale, see Supplementary file 2), to assess participants’ basic perceptions of the new drug administration system. Both the values^15^ and situation perceptions ^16^ scales are well-established and have been validated in previous research (see further details below). In the Phase 2, one-year follow-up, we added an 11-item questionnaire developed on the basis of participants’ responses to the open-ended questions in Phase 1, and three questions about their overall satisfaction with the new system (see details in our description of the study materials, below).

###  Study Materials

*Personal values* were measured using an abbreviated 10-item values scale,^17^ based on Schwartz theory of personal values.^15^ The theory’s validity has been demonstrated in extensive cross-cultural research (see Sagiv and Schwartz SH,^12^ Sagiv et al^18^ for a recent review). The measured values were: power, achievement, security, tradition, conformity, benevolence, universalism, self-direction, stimulation and hedonism (eg, conformity values: “Obeying social norms and expectations and avoiding actions that are likely to upset others. Being polite and self-disciplined. Honoring parents and elders”; see Supplementary file 1).

*Situation perceptions* were measured using the Situation Six scale, capturing categories that have been identified as basic dimensions through which people perceive and evaluate daily situations and events.^16^ The scale yields scores on six basic attributes of the life situations people encounter (in this case, the introduction of the new drug administration system). It provides information about the degree to which respondents perceive the situation as particularly positive or negative, familiar, demanding, odd, and straightforward.

*Attitudes towards the new system* were assessed in Phase 2 using an 11-item scale we developed, based on participants’ responses to the open-ended questions in Phase 1 (see Phase 1 results, below). The items pertained to both potential advantages (eg, provides more control) and disadvantages (eg, wastes time) of the new system (see below). Three additional items were introduced for assessing the overall satisfaction with the system (see all attitude items in Supplementary file 3).

###  Analyses

 We began the analyses of Phase 1 data with a content analysis of the two open-ended questions about participants’ views of the positive and negative implications of the new system.^19^ Two research assistants, blind to the goals of the current research, reviewed participants’ responses and used them to generate thematic categories. We then calculated descriptive statistics for the situation perception dimensions, for the nurses and physicians, Pearson and Spearman correlations between the most frequently noted responses (ie, pros and cons) to the new system and staff members’ basic perceptions of the system and personal values. For the Phase 2 data we first calculated descriptive statistics for each of the situation perceptions and the attitudes toward the new system (pros and cons), for nurses and physicians. We then conducted an exploratory factor analysis for the attitude variables and conduct a multiple regression analysis using the variables that yielded significant correlations with participants’ overall satisfaction with the new system. A possible mediation effect (ie, indirect effect) was assessed using Hayes’ Process Macro in SPSS 27.^20^ In addition, we calculated Pearson correlations between the factors obtained and both the situation perception dimensions and values.

## Results

###  Phase 1: Pre-implementation Evaluations of the New System

 Overall, 152 hospital physicians and nurses completed the questionnaire. Four responses with missing occupational group data, and five lacking material data were excluded. We thus remained with responses from 70 nurses (59 female) (mean age = 34.8 years, SD = 9.95) and 73 physicians (19 female) (mean age = 41.5 years, SD = 12.62) for the analyses. Average nurse tenure was 7.5 years (SD = 6.9) and average physician tenure was 9.5 years (SD = 10.2).

####  Content Analysis of the Open-Ended Questions

 The initial step was to conduct the content analysis of the open-ended questions about anticipated positive and negative implications of the new drug authorization system. A variety of anticipated implications were raised by both nurses and physicians (see Table 1). **Positive aspects:** A relatively large number of nurses and physicians expected that the new system would provide *improved monitoring* and *administration procedures*. More nurses than physicians (20 vs 7) noted that the new system would *reduce unnecessary use of antibiotics *and more modest numbers in both groups felt that it would lead to a *decrease in antibiotic resistance*. These reactions suggest that some respondents had a good understanding of the system’s goals and advantages. **Negative aspects:** Both nurses and physicians expressed concerns that the new system would *complicate the antibiotic administration process and waste time* and would *pose technical difficulties*. Overall, physicians tended to anticipate more negative effects than did nurses (56 versus 33), some worrying that thenew system* would harm physicians’ status and power* and yield *less cooperation with physicians*. A few were also concerned that the new system would *lack sufficient human judgement* and *harm new physicians’ training process*. Thus, many respondents were concerned about administrative or technical aspects of the process. Fewer raised concerns about the merits of the new system or its implications.

**Table 1:** Types and Frequency of the New System’s Implications as Appraised by Nurses and Physicians (Phase 1)

**Appraisal Type**	**Implication Category**	**Nurses (N = 70)**	**Physicians (N = 73)**
Positive	Improved monitoring and control	27	22
	Reduction in unnecessary antibiotics	20	7
	Improved procedure	13	12
	Less resistance to antibiotics	9	15
	Efficient	7	4
	Economic benefits	6	3
	Improved safety	3	2
	Better standardization	2	2
	More orderly process	2	6
	Decreased work load	2	1
	Innovative	0	1
Negative	Complicates process, wastes time	14	13
	Technical difficulties	8	4
	Less cooperation with physicians	4	9
	Harms physicians’ status and power	4	15
	Complicates nurses work	2	1
	Lack of human judgement	1	7
	Harms physicians’ training	0	5
	Lack of transparency	0	2

####  Quantitative Analyses

 We next examined the quantitative data (Tables 2 and 3) to identify the basic attributes of the new system and the associated types of personal perceptions and values. In Table 2 we provide descriptive statistics for the six situation perception dimensions for nurses and physicians. In Table 3 we include Pearson correlations of the key response categories (ie, pros and cons of the system) proposed with personal values and situation perception dimensions. Spearman correlations yielded practically the same results.

**Table 2:** Situation Perceptions by Occupation Group (Phase 1)

**Variable**	**Nurses**			**Physicians**		
	**N**	**Mean**	**SD**	**N**	**Mean**	**SD**
Situation perception dimensions (range from 1-5)						
Positivity	66	2.94	1.13	68	2.81	0.93
Familiarity	66	2.92	0.83	68	2.98	0.66
Demanding	66	2.93	1.00	68	2.73	0.88
Oddness	66	1.77	0.87	68	1.92	0.83
Straightforward	64	3.54	1.04	67	3.44	0.72
Negativity	64	1.60	0.90	67	1.68	0.90

Abbreviation: SD, standard deviation.

**Table 3:** Correlations Between Personal Values and Situation Perceptions and the Main Expectations From the New ASP (Phase 1, Physicians and Nurses Together)

	**Less Antibiotic Resistance**	**Less Unnecessary Antibiotics**	**Waste of Time**	**Less Cooperation With Physicians**	**Hurts Physicians' Power**
Personal values					
Power	0.08	-0.08	-0.06	**-0.21** ^*^	0.04
Achievement	0.04	0.03	0.00	0.03	0.16
Security	-0.11	0.06	-0.05	0.08	0.12
Tradition	-0.03	0.09	0.06	-0.04	**-0.19** ^*^
Conformity	-0.16	0.09	**-0.22** ^*^	0.13	**-0.22** ^*^
Benevolence	0.04	0.00	0.10	-0.08	0.06
Universalism	-0.12	0.12	0.08	0.02	-0.12
Stimulation	0.04	**-0.22** ^**^	0.03	0.05	0.00
Self-Direction	0.07	0.04	0.05	0.08	0.15
Hedonism	0.05	-0.04	0.03	0.16	0.11
Situation perception dimensions					
Positivity	-0.16	-0.02	-0.17	0.02	-0.11
Familiarity	-0.07	-0.07	-0.07	0.10	0.01
Demandingness	0.14	0.12	**0.20** ^*^	0.10	0.00
Oddness	0.05	0.03	0.04	-0.04	0.02
Straightforwardness	0.01	-0.01	0.00	0.10	-0.01
Negativity	0.06	0.02	-0.02	-0.05	-0.13

* *P* <.05 (2-tailed), ** *P* <.01 (2-tailed).

####  Situation Perception 

 Situation perception scores indicate that both nurses and physicians perceived the new antibiotic administration system as quite *straightforward* (Mean values on a 5-point Likert scale: nurses 3.54, physicians 3.44), and as not *odd* (nurses 1.77, physicians 1.92) nor particularly *negative* (nurses 1.60, physicians 1.68). There were no significant differences between nurses’ and physicians’ ratings of these six perception dimensions.

####  Values and Attitudes Towards the New System

 See Table 3, top section, focusing on the most frequently noted positive and negative implications. In this analysis nurses’ and physicians’ responses were taken together. Respondents who valued conformity (ie, motivated to comply with norms and expectations) were less likely to express concerns that the new system would waste time (r = -0.22, *P* < .05). These respondents, as well as those who valued tradition (ie, motivated to maintain the status quo), were less likely to view the new system as harming physicians’ status (r = -0.22, -0.19, *P* < .05). Respondents who emphasized power were less likely to worry about physicians’ cooperation with the new system (r = -0.21, *P* < .05), and those who emphasized stimulation were less likely to anticipate a decrease in unnecessary antibiotics (r = -0.22, *P* < .05). All of these effects were significant, although relatively weak (ie, *P* < .03).

####  Situation Perceptions And Attitudes Towards the New System

 Notably, perceiving the new system as demanding was associated with expressing concerns that the new system will waste time (r = 0.20, *P* < .05) (Table 3, bottom section).

 In sum, the respondents in Phase 1 identified both positive and negative aspects of the new system. Whereas physicians and nurses identified similar positive aspects, physicians expressed more concerns than nurses. Overall, personal values and perception of the situation had little effect on the attitudes towards the new system at this early stage.

###  Phase 2: One Year Post-implementation 

 In this phase responses were obtained from 46 nurses and 66 physicians (Table 4), not all of whom had participated in Phase 1.

**Table 4:** Study Variables by Occupation Group (Phase 2)

**Variable**	**Nurses**			**Physicians**		
	**N**	**Mean**	**SD**	**N**	**Mean**	**SD**
Gender (Male = 0, Female = 1)	44	0.80	0.41	64	0.36	0.45
Age	46	37.11	10.20	62	41.52	9.95
Situation perception dimensions (range from 1-5)						
Positivity	41	3.20	0.94	66	2.68	0.97
Familiarity	40	3.26	0.77	65	2.95	0.89
Demanding	40	2.58	1.01	65	2.59	1.01
Oddness	39	2.01	0.90	65	1.69	0.81
Straightforward	39	3.74	0.89	65	3.21	0.84
Negativity	39	1.62	0.88	65	1.53	0.88
Attitudes toward new system (system leads to…; range from 1-5)						
Greater control	43	4.16	0.97	64	4.13	0.90
Less unnecessary medications	43	3.74	1.22	64	3.69	1.14
Better distribution process	43	4.14	0.91	64	3.08	1.10
Less antibiotic resistance	42	3.81	1.29	64	3.45	1.04
Greater efficiency	42	4.02	0.87	64	2.95	1.21
Financially beneficial	41	3.80	1.21	64	3.64	1.01
Waste of time	41	2.12	0.87	64	3.11	1.18
Harm to physicians’ status	42	1.79	0.98	64	2.19	1.11
Technical difficulties using system	41	2.54	1.27	63	2.79	1.23
Insufficient physician cooperation	40	2.63	1.25	63	2.43	0.96
ID consultants should not have authority to prescribe antibiotics	42	2.02	1.49	64	2.22	1.13
Prefer former system	42	1.69	1.00	62	1.55	0.94
High satisfaction with system	40	3.98	0.80	62	3.23	1.03
Will recommend to other hospitals	41	4.12	0.93	62	3.24	1.15

Abbreviations: SD, standard deviation, ID, infectious diseases.

####  Overall Satisfaction With the New System

 One year following its implementation, nurses were clearly satisfied with the new system (Mean = 3.98, SD = 0.80), significantly more so than the physicians (Mean = 3.23, SD = 1.03; t = 4.2, *P* < .01). This corresponds with our observation in the first phase that physicians tended to report more concerns than did the nurses.

####  Positive and Negative Aspects of the New System

 An exploratory factor analysis of the reactions to the new system revealed three attitude factors associated with the level of satisfaction. One set of reactions involved *benefits* for patients or the hospital’s functioning. These included “greater control and monitoring,” “minimizing the distribution of unnecessary medicine,” “minimizing drug tolerance,” and “financial benefits to the hospital.” Respondents tended to acknowledge these benefits of the new system (Mean = 3.78, SD = 0.91). The second factor of reactions involved improvements that have to do with new system’s *efficiency* (ie, “better drug distribution process,” “greater efficiency”). Nurses largely acknowledged these advantages (Mean = 4.06, SD = 0.82), substantially more so than did the physicians (Mean = 3.02, SD = 1.04). The third set of reactions involved *problems* or challenges with the new system, including “more technical difficulties,” “wasting time,” “insufficient physician cooperation,” and perceived “harm to physicians’ status.” Agreement that these problems exist tended to be moderate-low (Mean = 2.48, SD = 0.85).

 The three factors were strongly inter-correlated, such that those who tended to identify one type of advantage tended to identify the other type as well, and were less likely to be concerned about the system’s potential problems. The indexes of the three factors strongly correlated with participants’ satisfaction with the new system (r = 0.56, 0.72, -0.56 for benefits, efficiency, and problems, respectively, all *P* < .01). In a multiple regression analysis, the three factors together explained 58% of the variance in the overall satisfaction.

####  Situation Perceptions

 After using it for a year, both nurses (Mean = 2.58) and physicians (Mean = 2.60) perceived the new system as somewhat demanding, but less so than anticipated before implementation in Phase 1 (t = 1.86, *P* = .06). Nurses viewed the system as more positive (Mean = 3.20) and straightforward (Mean = 3.74) than did the physicians (Mean = 2.68 and 3.21, respectively, both t >2.74, *P* < .01). Nurses also tended to view it as slightly more familiar (Mean = 3.26 vs. 2.95, t = 1.89, *P* = .06) and more odd (Mean = 2.01 vs. 1.69, t = 1.80, *P* = .08). Neither nurses nor physicians viewed the new system as particularly negative (Mean = 1.62 and 1.53, respectively).

####  Situation Perceptions and Attitudes Toward the New System

 All three attitude factors and the overall satisfaction were correlated with the basic perceptions of the new system (top section of Table 5). As could be expected, benefits of the new system and its efficiency were correlated positively with perceiving the system as positive and straightforward, and negatively with perceiving the system as negative, demanding and odd. Effect sizes were generally moderate (.3 < *P* < .5), with a few exceptions, such as the strong correlation between identifying problems with the system and its perceived demandingness. Interestingly, perceiving the new system as familiar was positively (weakly) correlated with the efficiency factor, suggesting that as medical staff gain experience with the system, they also perceived it as (and it may have actually become) more efficient.

**Table 5:** Correlations Between Attitude Factors and Situation Perceptions and Personal Values (Time 2)

	**Benefits**	**Efficiency**	**Problems**	**Overall Satisfaction**
Situation perception dimensions				
Positivity	0.33^**^	0.56^**^	-0.25^*^	0.51^**^
Familiarity	0.02	0.24^*^	0.02	0.13
Demandingness	-0.23^*^	-0.30^**^	0.62^**^	-0.41^**^
Oddness	-0.32^**^	-0.17	0.40^**^	-0.30^**^
Straightforwardness	0.28^**^	0.39^**^	-0.28^**^	0.39^**^
Negativity	-0.30^**^	-0.26^**^	0.45^**^	-0.42^**^
Personal values				
Power	-0.10	-0.03	0.18	-0.08
Achievement	-0.14	-0.09	-0.07	-0.13
Security	0.07	-0.01	-0.10	-0.04
Tradition	-0.02	0.01	0.12	-0.01
Conformity	0.25^**^	0.16	-0.02	0.23^*^
Benevolence	0.15	-0.04	-0.13	-0.06
Universalism	-0.02	-0.06	0.04	-0.10
Stimulation	0.07	0.15	-0.07	0.15
Self-direction	-0.12	-0.15	0.01	-0.19
Hedonism	-0.11	0.00	-0.11	0.16

* *P* <.05 (2-tailed), ** *P* <.01 (2-tailed).

####  Values and Attitudes Toward the New System

 We found a number of significant correlations between respondents’ personal values and their overall and specific attitudes towards the new system (bottom section of Table 5). Specifically, conformity values—expressing the motivation for compliance with social norms and expectations—were positively correlated with overall satisfaction with the new system. Emphasizing these values was also positively correlated with reports of the benefits of the system. When included together with the three attitude factors (ie, benefits, efficiency, and problems) in a regression analysis for predicting the overall satisfaction with the new system, conformity values had a marginally significant effect (t = 1.92, *P* < .06). Thus, participants who value compliance with norms and expectations were more likely express satisfaction with the new system even beyond their perceptions of the system’s benefits.

 We conducted another multiple regression analysis to predict overall satisfaction, this time including as predictors the six variables that yielded significant correlations with overall satisfaction (in Table 5). All of the predictors remained significant (*P* < .05) with the exception of oddness perceptions and conformity values. Given that conformity values are conceptually associated with how straightforward events are perceived to be, we followed by testing the possibility that straightforwardness perceptions mediate the effect of conformity values on the overall satisfaction with the system. The mediation effect (ie, indirect effect) was significant (standardized indirect effect = 0.07, standard error = 0.03, bootstrapping lower level confidence interval = 0.008, bootstrapping upper level confidence interval = 0.158). Thus, conformity values predicted perceptions of the new system as straightforward, which, in turn, predicted satisfaction with the new system.

## Discussion

 Interventions of antimicrobial stewards in the processes of changing and improving antibiotic use may be regarded as beneficial (eg, involving a more orderly process and more shared responsibility) or as an intrusive process that interferes with physicians’ autonomy and authority. Evaluations of any intervention, as well as antimicrobial stewardship, depend, at least in part, on the manner in which the new system is introduced to stakeholders such as nurses and physicians, as well as on their personal motivations.^21^ Thus, to be effective, antimicrobial stewardship interventions should account for psycho-social factors.^6,13,22^

 Antimicrobial stewardship leads to a sustained reduction in antimicrobial use,^23-25^ and has been used in our hospitals for decades. The current change was the driving force for a ‘bottom-up’ analysis of emergent themes. Given the importance of physicians’ and nurses’ attitudes for the success of new ASPs, our aim was to uncover these attitudes and the basic perceptions and values that are associated with them.

 When the new system was first introduced, staff members were concerned that the new system would be inefficient and would restrict physicians’ autonomy and status. In addition, they perceived it as demanding, perhaps reflecting the newly introduced requirement to specify the correct indication for the required antibiotic treatment and address antimicrobial stewards’ consults. A year after the implementation, however, both nurses and physicians perceived the system as less demanding than anticipated. Moreover, the degree to which the system was perceived as familiar was associated with perceiving it as more efficient.

 In retrospect, the system’s benefits were already anticipated in Phase 1, noted by the improved efficiency of the antibiotic administration process. Thus, while the respondents were concerned with inefficiency, they also expected the new system to improve the process. Other reported advantages included benefits to the hospital (ie, financial), patients (no unnecessary drugs) and society at large (less antibiotic resistance). Perceptions of these benefits became even more pronounced a year after the system’s introduction. Overall, the sentiment toward the new system in Phase 2 was positive, highlighting its perceived benefits, as efficient and overall satisfactory.

 Naturally, greater acknowledgement of the integrated ASP’s benefits was associated with greater overall satisfaction with it. In line with the general insights about the process of introducing changes and innovations,^26^ clearly articulating the system’s benefits was a key aspect of its introduction. Awareness to the anticipated concerns about the system, especially about its demandingness and inefficiency, should also inform the manner in which the change is introduced, so as to alleviate these concerns, thus setting the stage for a smooth implementation process. A balanced presentation may also benefit others; conducting a fruitful discourse between ID consultants and prescribers and involving medical students in the process is another way of educating future stakeholders.

 Our findings also point to interpersonal differences in stakeholders’ responses.^27^ Nurses and physicians who value conformity, and are thus predisposed to obey rules and expectations, tended to express greater satisfaction with the new system than those lacking such predispositions. This implies that the new system’s introduction was attributed to the legitimate authorities within the medical center. This is consistent with previous research showing that those who value conformity tend to support management’s initiatives for organizational change.^28^ However, those who value conformity often object to such change initiatives because of their inherent preference for the status quo. In the present study, the motivation to accept the institutional action appears to have predominated over the disinclination to breaking the status quo. It predicted perceptions of the new system as straightforward, which in turn predicted satisfaction with the new system. Highlighting stakeholders’ involvement in introducing the new system could therefore strengthen staff members’ buy in to the new system.

###  Study Limitations

 Being a case study in one locale, the degree to which our findings could be generalized to other medical centers is unclear. Future research should therefore aim to assess the implementation in additional centers in a variety of countries and cultures. In addition, given our study’s design, we cannot determine the causal nature of the relationships among perceptions, values and the attitudes toward the system. Nevertheless, even without identifying the causality involved, learning about the associations between these factors is useful for understanding staff members’ responses to the new ASP. Future research could aim to track the same participants’ responses over time and would thus be better positioned to address the causal nature of the relationships.

 In conclusion, we provide an example of a successful implementation of a change in an EMR based stewardship, in which the final decision is left to the ID stewards. Our findings are important for ASP stakeholders. First, we demonstrate that the new system can be viewed positively by key stakeholders, especially by those who are motivated to comply with norms and expectations. Initial concerns diminished, and perceived benefits were maintained. It is therefore important to highlight the system’s benefits early on, in particular those involving increased order and efficiency.

## Acknowledgements

 The authors thank all participating physicians and nurses. We thank Prof. Colin Block for his useful comments on an earlier version of the manuscript.

## Ethical issues

 The study was approved by the institutional review boards (approval 0557-16-HMO) of the Hebrew University and Hadassah-Hebrew University Medical Center, Jerusalem, Israel.

## Competing interests

 Authors declare that they have no competing interests.

## Authors’ contributions

 All authors were involved in the design, execution and interpretation of results of the study, and in the writing of the manuscript.

## Funding

 This work was supported by the Israeli Science Foundation (grant 847/14, L.S) and the Recanati Fund of the Hebrew University’s School of Business Administration (SO and LS).

## Supplementary files


Supplementary file 1. Personal Values Scale.

Supplementary file 2. Situation Perceptions Scale.

Supplementary file 3. Items for Assessing Attitudes Toward the New ASP in Phase 2.
