# Supplementary material for: Nurses’ and Physicians’ Responses to a New Active Antimicrobial Stewardship Program: A Two-Phase Study of Responses and Their Underlying Perceptions and Values
Source: Int J Health Policy Manag. 2022 May 18;11(12):2982–9. doi: 10.34172/ijhpm.2022.6557 (PMC10105176; doi:10.34172/ijhpm.2022.6557)
Supplement: Supplementary file 1 — Personal Values Scale. [file ijhpm-11-2982-s001.pdf]

Article title: Nurses' and Physicians' Responses to a New Active Antimicrobial Stewardship Program: A Two-Phase Study of Responses and Their Underlying Perceptions and Values

Journal name: International Journal of Health Policy and Management (IJHPM)

Authors' information: Jacob Strahilevitz[1,2]*, Shaul Oreg[3], Ran Nir Paz[1,2], Lilach Sagiv[3]

[1]Department of Clinical Microbiology and Infectious Diseases, Hadassah-Hebrew University Medical Center, Jerusalem, Israel.

[2]Faculty of Medicine, The Hebrew University, Jerusalem, Israel.

[3]School of Business Administration, The Hebrew University, Jerusalem, Israel.

(*Corresponding author: jstrahilevitz@hadassah.org.il)

Supplementary file 1. Personal Values Scale

Below is a list of ten values. For each value, please state how important it is for you, by writing the appropriate score in the column next to the value.

-1	0	1	2	3	4	5	6	7
Opposed to my values	Not important			Important			Very important	Of supreme importance

**The value**	**Importance**
Caring about the welfare of people with whom one is in personal contact. Being responsible, loyal, honest and forgiving.	___
Being independent in thought and action. Being open to novelty and change. Living freely, expressing curiosity and creativity.	___
Understanding and accepting all people, caring for their welfare and equality. Being intellectually and emotionally open to the environment and caring for nature.	___
Living safely, in an organized, stable place. Keeping one's family secure and healthy.	___
Obeying social norms and expectations and avoiding actions that are likely to upset others. Being polite and self-disciplined. Honoring parents and elders.	___
Aspiring for impressive achievements. Demonstrating competence, excellence and personal success.	___
Having fun and sensuous gratification. Enjoying life, being self-indulgent.	___
Experiencing constant change, excitement, novelty, and challenge in life. Being daring, living a varied and exciting life.	___
Preserving the status-quo, doing things that are familiar and known. Being humble and moderate. Respecting the customs of the traditional culture or religion provide.	___
Having social status and prestige, and winning influence over other people. Being rich and controlling resources.	___
