# Supplementary material for: Nurses’ and Physicians’ Responses to a New Active Antimicrobial Stewardship Program: A Two-Phase Study of Responses and Their Underlying Perceptions and Values
Source: Int J Health Policy Manag. 2022 May 18;11(12):2982–9. doi: 10.34172/ijhpm.2022.6557 (PMC10105176; doi:10.34172/ijhpm.2022.6557)
Supplement: Supplementary file 2 — Situation Perceptions Scale. [file ijhpm-11-2982-s002.pdf]

Article title: Nurses' and Physicians' Responses to a New Active Antimicrobial Stewardship Program: A Two-Phase Study of Responses and Their Underlying Perceptions and Values

Journal name: International Journal of Health Policy and Management (IJHPM)

Authors' information: Jacob Strahilevitz[1,2]*, Shaul Oreg[3], Ran Nir Paz[1,2], Lilach Sagiv[3]

[1]Department of Clinical Microbiology and Infectious Diseases, Hadassah-Hebrew University Medical Center, Jerusalem, Israel.

[2]Faculty of Medicine, The Hebrew University, Jerusalem, Israel.

[3]School of Business Administration, The Hebrew University, Jerusalem, Israel.

(*Corresponding author: jstrahilevitz@hadassah.org.il)

Supplementary file 2. Situation Perceptions Scale

	To what degree do each of the following characterize the change [i.e., the implementation of the new system]				
	**1**<br>**Not at all characterizes**	**2**	**3**	**4**	**5**<br>**Very much characterizes**
1. Amazing	1	2	3	4	5
2. Wonderful	1	2	3	4	5
3. Great	1	2	3	4	5
4. Expected	1	2	3	4	5
5. Constant	1	2	3	4	5
6. Typical	1	2	3	4	5
7. Complicated	1	2	3	4	5
8. Exhausting	1	2	3	4	5
9. Challenging	1	2	3	4	5
10. Provocative	1	2	3	4	5
11. Silly	1	2	3	4	5
12. Crazy	1	2	3	4	5
13. Objective	1	2	3	4	5
14. Focused	1	2	3	4	5
15. Concrete	1	2	3	4	5
16. Horrifying	1	2	3	4	5
17. Horrible	1	2	3	4	5
18. Shameful	1	2	3	4	5
