# Supplementary material for: Nurses’ and Physicians’ Responses to a New Active Antimicrobial Stewardship Program: A Two-Phase Study of Responses and Their Underlying Perceptions and Values
Source: Int J Health Policy Manag. 2022 May 18;11(12):2982–9. doi: 10.34172/ijhpm.2022.6557 (PMC10105176; doi:10.34172/ijhpm.2022.6557)
Supplement: Supplementary file 3 — Items for Assessing Attitudes Toward the New ASP in Phase 2. [file ijhpm-11-2982-s003.pdf]

Article title: Nurses' and Physicians' Responses to a New Active Antimicrobial Stewardship Program: A Two-Phase Study of Responses and Their Underlying Perceptions and Values

Journal name: International Journal of Health Policy and Management (IJHPM)

Authors' information: Jacob Strahilevitz[1,2]*, Shaul Oreg[3], Ran Nir Paz[1,2], Lilach Sagiv[3]

[1]Department of Clinical Microbiology and Infectious Diseases, Hadassah-Hebrew University Medical Center, Jerusalem, Israel.

[2]Faculty of Medicine, The Hebrew University, Jerusalem, Israel.

[3]School of Business Administration, The Hebrew University, Jerusalem, Israel.

(*Corresponding author: jstrahilevitz@hadassah.org.il)

Supplementary file 3. Items for Assessing Attitudes Toward the New ASP in Phase 2

The new system leads to:…

1. Greater control
2. Less unnecessary medications
3. Better distribution process
4. Less antibiotic resistance
5. Greater efficiency
6. Financially beneficial
7. Waste of time
8. Harm to physicians' status
9. Technical difficulties using system
10. Insufficient physician cooperation
11. infectious diseases consultants should not have authority to prescribe antibiotics

Questions for assessing overall satisfaction with the new ASP:

12. I am very satisfied with the new system
13. I would recommend to other hospitals to use the new system
14. I prefer the former system (reverse-scored).
